# Assessment of Registered Clinical Trial Designs: Comparison of L-Arginine and/or L-Citrulline Interventions for Hypertension

**DOI:** 10.3390/ph17040477

**Published:** 2024-04-08

**Authors:** Ashley Brett Hillsley, Craig Steven McLachlan

**Affiliations:** Center for Healthy Futures, Torrens University Australia, Surry Hills, NSW 2010, Australia; ahillsley@torrens.edu.au

**Keywords:** L-arginine, blood pressure, clinical trials, intervention

## Abstract

Background: L-Arginine (Arg) is an essential amino acid and a precursor for the synthesis of vascular nitric oxide, while L-Citrulline is a non-essential amino acid substrate for increasing L-arginine. Both L-arginine and L-Citrulline in translational studies may acutely lower the blood pressure. Current meta-analysis for L-arginine or L-Citrulline interventions in blood pressure have identified significant heterogeneity. Clinical trial evidence for L-arginine or L-Citrulline in chronic blood pressure reduction in the general population requires an examination of trial designs, as not all translational studies may have influenced vascular reactivity. Our aims are to explore whether L-arginine and L-citrulline intervention trials in chronic blood pressure consider standardized end points relevant to the general adult populations. Methods: A step-wise search on clinicaltrials.gov, the U.S. Library of Medicine registry for clinical trials, was performed including the following keyword search parameters: “completed” “L-Citrulline” “L-arginine” trial”, and “adults”, involving “blood pressure” reduction as a primary end point in adult humans. Results: Of the forty-four completed trials, only five were included for analysis. Following the careful evaluation of trial design, we observed heterogeneity across participant inclusion criteria (population sample size, age range, sex), interventional design (dosages, duration), and primary outcomes, measured with respect to changes in diastolic or systolic blood pressure. Conclusion: In conclusion, there is a lack of robust trial design evidence to suggest that L-arginine or L-Citrulline, based on current RCTs in the general population, have an overall positive effect on vascular endothelial reactivity and a beneficial chronic blood pressure-lowering effect. Indeed, conclusions drawn from human meta-analysis studies have been heterogenous between studies, which may be attributed to study design heterogeneity, including differences in sample population, age, and blood pressure at the time of entry. Inconsistencies in the study design poses a challenge for systematic reviews and meta-analysis to accurately assess the effect size and impact of L-arginine or L-citrulline on both systolic and diastolic blood pressure.

## 1. Introduction

Vascular nitric oxide (NO) is released from endothelium and has a direct vasodilator effect on healthy human arterial tissues. L-arginine is an essential amino acid, as well as a precursor to endothelial nitric oxide synthase (eNOS) and the main precursor of nitric oxide (NO) released from vascular endothelium [1,2]. In acute human studies, oral supplementation of L-arginine results in the improvement of endothelium-dependent forearm vasodilation in subjects with pre-existing hyper lipidemia [3]. Subjects with pre-existing high cholesterol and other cardiovascular or metabolic risk factors are known to have both reductions in vascular reactivity and altered eNOS activity [4]. Additionally, in active smokers, both the uncoupling of eNOS activity and reduced vascular reactivity have been observed [5,6]. The acute effects of intravenous L-arginine have demonstrated immediate improvements in terms of vascular relaxation. Thus, it has been suggested that L-arginine likely has a direct modifying effect on endothelium-dependent vasoreactivity, in addition to its positive effects on platelets and leucocytes [7].

A recent meta-analysis examined the assessed trials of L-arginine’s effect on human blood pressure; however, the examined populations were heterogenous with respect to a focus on specific pre-existing conditions and dosing [8]. There has been a lack of L-arginine blood pressure clinical trials focusing on the general population.

Despite the use of L-arginine in translational studies, the heterogeneity in terms of design elements and outcomes between and within human randomized control trials (RCT) poses a significant challenge for meta-analysis inclusion. For example, Shiraseb et al. (2022) conducted a systematic review and meta-analysis where L-arginine supplementation was found to have modest effects on pooled systolic and diastolic blood pressure across studies [8]. Nonetheless, the authors also noted that there were design issues related to the published studies, which included a lack of information regarding randomization efficiency and withdrawal information. Interestingly, study sample sizes were small, suggesting futility in controlling for disease confounder’s and other environmental and demographic factors. Additionally, across the studies in the meta-analysis, it was noted that individuals with varying degrees of health statuses were included, thus contributing to a heterogeneous sample for both systolic and diastolic outcomes between and within studies [8].

Endothelial function regulated by nitric oxide via L-arginine is somewhat complex with respect to the biochemical interactions that can affect the efficacy of L-arginine [9,10]. Examples include dose, absorption, duration, biochemical pathways, and pathological aging, which may interfere with blood pressure challenges, such as vascular stiffening associated with co-morbidities that include diabetes, metabolic syndrome, fatty liver disease, lipid disorders, obesity, arthritis, and prior stroke [11,12,13]. The complexity of studies targeting pharmacological interventions that are proposed to work via improving vascular reactivity, and thereby improve blood pressure control, require the standardization of study designs. Indeed, study protocols for L-arginine should ideally encompass appropriate inclusion and exclusion criteria and primary and secondary outcome measures. Such variables from a pharmacological perspective should include the dosage and frequency of L-arginine administration, as the dosage has been shown to influence vascular reactivity in human studies, the route of administration, the duration of intervention, single or multi-compound intervention, the method of blood pressure measures, the interval of repeated blood pressure measures, co-morbidities, and population demographic.

The World Health Organization (WHO) in 2004 called for the establishment of “a network of international clinical trials registers to ensure a single point of access and the unambiguous identification of trials”, and, furthermore, the WHO during the 58th World Health Assembly adopted Resolution WHA58.22, which stated that all clinical trials designs should “...link to clinical trials registers in order to ensure a single point of access...”. Trial information must contain minimum data that is relevant to the conduct of RCTs [14,15]; typically, the trials are deposited on ClinialTrials.gov. The clinicaltrials.gov database covers 220 countries and includes key information categories regarding study description, study design, details of interventions, primary and secondary outcome measures, eligibility criteria, contacts, and whether the trial is commencing or ongoing or completed. Despite clinicaltrials.gov being the gold standard for the registration of trials, not all RCTs are registered. Additionally, not all completed registered trials have had their trial results published in academic journals. Additionally, information for registered trials might be incomplete. Henceforth, meta-analysis will not have available unpublished studies, and we are not aware of any that include registry trial data. Thus, we aim to interrogate and identify variations in the trial designs of chronic L-arginine interventions in blood pressure, and to assess the clinical end points and overall trial designs using clinicaltrials.gov.

## 2. Results

**Figure 1:**
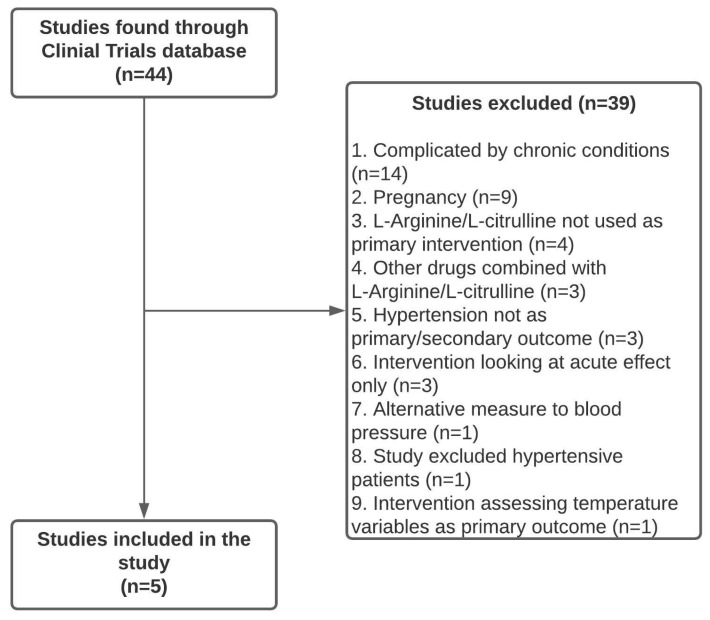
Flowchart of clinical trial selection process for inclusion in this study.

In this work, 44 completed clinical trials with L-Arginine were initially identified on the clinical trials database. Of the 44 trials identified, 39 were excluded. These were excluded due to subpopulations that focus on specific chronic conditions (n = 14), pregnancy (n = 9), L-Arginine/L-Citrulline not being used as the primary intervention (n = 4), other drugs combined with L-Arginine/L-Citrulline (n = 3), hypertension not utilized as a primary or secondary outcome (n = 3), the intervention only investigation the acute effect (n = 3), alternative measures for blood pressure (n = 1), the study excluded hypertensive patients (n = 1), and the intervention assessing temperature variables as a primary outcome (n = 1). The remaining clinical trials (n = 5) examined L-Arginine/L-Citrulline interventions in individuals over the age of 18 with blood pressure as an assessed outcome at the end of the trial (Figure 1).

### 2.1. Characteristics of the Clinical Trial

Of the five clinical trial groups included from clinicaltrials.gov, there was no trial that met our inclusion criteria which also included a specific chronic disease subgroup, e.g., T2DM. Specifically, one RCT study group (NCT03433963) included ageing individuals with elevated blood pressure (systolic blood pressure between 120–160 mmHg, and diastolic blood pressure between 65–100 mmHg without current medication management). A further RCT (NCT01185041) had a population sample of ageing individuals with a combination of obesity and pre-existing hypertension. In another RCT (NCT02392767), the trial population group included moderate hypertension with hyperhomocysteinemia; another RCT study (NCT02143817) included ageing postmenopausal obese women. In the final included RCT (NCT00137124), the population groups included those perceived as healthy and those with a possible genetic endothelial dysfunction, being known offspring to hypertensive parents. In summary, the characteristics for the populations examined were not selected for a specific chronic disease in the clinicaltrials.gov register.

**Figure 2:**
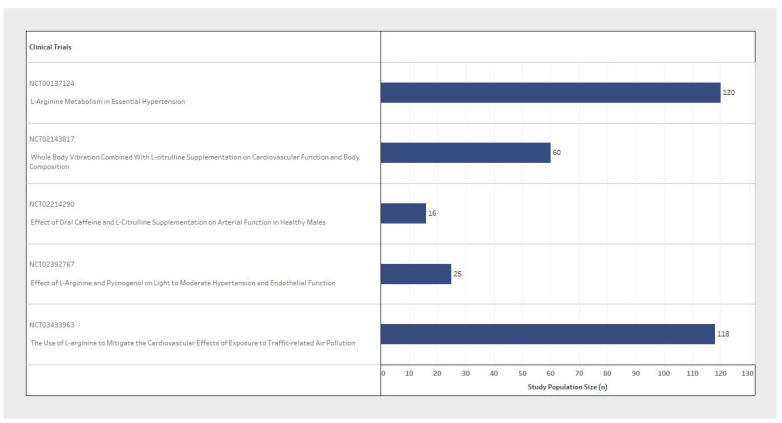
Comparison of the participant enrolment size between clinical trials.

**Figure 3:**
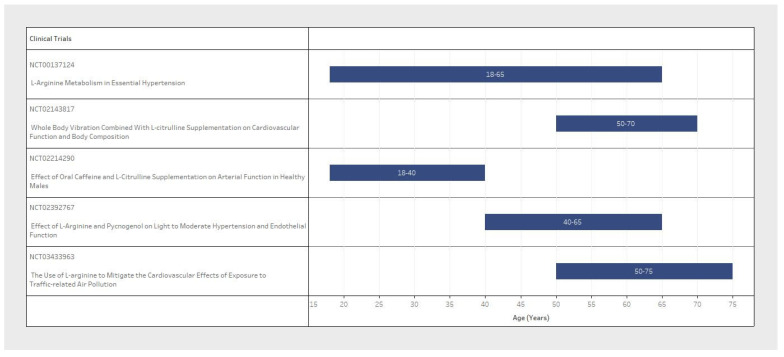
Comparison of the participant age range between clinical trials.

**Figure 4:**
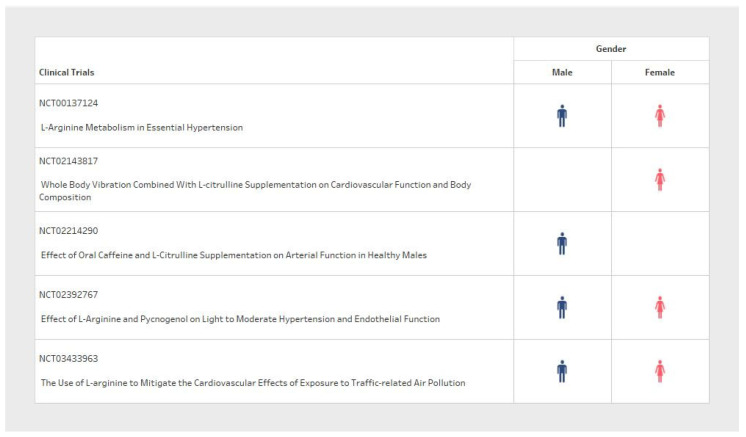
Comparison of the participant sex between clinical trials.

Sample size and participant inclusion criteria for each of the five studies varied significantly between clinical trial design (Table 1). The population sample size ranged from 16 to 120 participants (Figure 2), with considerable variability in the basic demographic information. The minimum participant age also varied from 18 to 75 years old between trials (Figure 3). With respect to sex variables, one study included only male participants dosed with L-Arginine (Figure 4, blue only); another study was a post-menopausal female-only sample population dosed with L-Citrulline (Figure 4); and three RCTs included both male and female participants dosed with L-Arginine (Figure 4). Furthermore, despite the design of these studies with the eventual reported outcome of chronic blood pressure reduction, three of the five studies did not explicitly state a diastolic blood pressure range, and two did not state a systolic blood pressure range for participant selection. Furthermore, one of the three studies that did include a blood pressure range for selection criteria had no specified minimum blood pressure cut-off for both systolic and diastolic. Only two studies reported an upper and lower blood pressure limit to their inclusion criteria (Table 1, inclusion criteria). This means that all participants could potentially be hypertensive within these trial designs.

L-Arginine or L-Citrulline were administered as a direct oral supplement (n = 5) (Table 1, intervention). For L-Arginine, the oral dosage ranged from 2400–3000 mg 2–3 times a day. The frequency of dosing for L-Arginine ranged from 14 days to 28 days. By comparison, only one L-Citrulline intervention included information regarding the dosage. This was 75 mg per dose, 8 times a day, over 7 days.

Primary outcomes and the number of outcomes varied across trials (Table 1, outcomes measured). While all trials measured included blood pressure measures in some form, only three of the five clinical trials listed markers of blood pressure (e.g., changes in acute brachial systolic and/or diastolic blood pressure or ambulatory or central/aortic blood pressure measurements) as a trial primary outcome. Types of blood pressures assessed included a combination of brachial ambulatory blood pressure (n = 5), central/blood pressure (n = 3), resting brachial blood pressure measurement (n = 2), and ankle blood pressure (n = 2).

### 2.2. Comparison of Trials with Meta-Analysis

We identified four meta-analyses that overlapped for the years of our included trials from clinicaltrials.gov [16,17,18,19]. One meta-analysis was removed from consideration due to retraction [19]. The clinicaltrials.gov trial elements identified in our study were compared to those included in the three published meta-analyses [16,17,18]. It was found that only one of the forty-four RCTs (before exclusion) we identified in clinicaltrials.gov overlapped with the studies included in the three pooled meta-analyses. Interestingly, aside from the one study we included, there were no other trials included in the pooled meta-analysis originally derived from clinicaltrials.gov (Figure 1). Hence, these three pooled meta-analyses did not include the remaining forty-three RCTs identified in our study, and the eighteen RTCs included in the meta-analyses were not registered on clinicaltrials.gov (Figure 1, studies excluded). The three meta-analyses had different focuses, where two meta-analysis focused on L-Citrulline, and one focused on L-arginine. None of the RCT studies in the L-arginine meta-analysis overlapped with the L-Citrulline meta-analysis we examined. Across the two L-Citrulline meta-analyses, five studies overlapped. Interestingly, only one of these meta-analyses included the RCT that was identified in clinicaltrias.gov and met our inclusion criteria.

## 3. Discussion

In this study, we aimed to explore trial designs for registered RCTs in clinialtrials.gov for studies that targeted the general population for blood pressure interventions using L-arginine or L-Citrulline. Variations in clinical trial blood pressure end points were evident; this is perplexing as the trials that met our inclusion criteria did not evaluate age-related reductions in blood pressure with either a compound or a physiological basis for this reduction.

For example, our analysis of readily available trial design information deposited in clinicaltrials.gov identified differences in inclusion criteria based upon a narrow age range, sex, metabolic syndrome, sample size, and interventions (dosage and interval). The outcome measures did not always include measures of arterial stiffness or measures of vascular reactivity in order to demonstrate any improvements in stiffness or vascular reactivity. Pre-defined screening for normative population data was carried out; despite this, aging is known to increase resistant systolic blood pressure due to arterial stiffening [20,21,22], and it is unknown whether either L-arginine or L-citrulline could reverse arterial stiffening, thereby improving arterial compliance.

Our primary finding was that not all registered trials provided fundamental inclusion and exclusion criteria for the same blood pressure end points, which would be necessary for the cross-comparison of baseline and outcome assessments. The studies included in our analysis lacked complete trial design information in many cases, suggesting the difficulty of using this evidence to determine if either L-arginine or L-Citrulline administration is effective for chronic blood pressure reduction or whether the reduction in blood pressure with a short dosing period is sustained. This would be especially apparent if one tried to pool these five studies for a mean effect (from the five studies we identified from clinical trials.gov).

Furthermore, the small sample size of these studies, ranging from 16–120 participants (per study), is likely to lead to heterogeneous outcomes. Heterogenous outcomes have been reported in meta-analysis studies and in umbrella reviews (of meta-analysis reviews) for L-arginine and the effects on blood pressure reduction [20]. Given the dosing and duration variability, coupled with the variability in sample size, it is difficult to draw strong conclusions for sustained blood pressure changes in a normative population sample.

We observed differences for sex allocation across trials where three out of five studies examined both sexes, whereas one out of five studies exclusively included men and one study only included women. Sex differences are a well-recognized biological factor in hypertension in both humans and rodent models, partly attributed to sex hormone levels, which can also change with age. For example, estrogen is associated with nitric oxide (NO) production [23,24]. Hence, as well as sex, age is also a factor, as women have significantly reduced estrogen and L-arginine levels during midlife pre-menopause and menopause; this could explain why a more significant blood pressure response may be seen with L-arginine in middle-aged women, where L-arginine may compensate for the reduced estrogen effects, or due to the L-arginine levels in increasing vascular nitric oxide [25]. Indeed, there is evidence to also suggest that post-menopausal blood pressure is increased in women [26,27]. Interestingly, estrogen replacement therapy has not been shown to decrease blood pressure in some studies [28]. In aging rats, increases are associated with a reduction in L-arginine and an excretion of NO metabolites [29]. Thus, it is also possible that the effect of age on other components of NO is important in the regulation of blood pressure.

Intriguingly, to utilize central systolic blood pressure in trials for novel blood pressure interventions is not standard practice. However, several trials we included also used central systolic blood pressure as a primary or secondary end point to observe differences in the chronic administration of therapeutic doses of L-arginine or L-citrulline. However, it is also known that large vascular stiffness that contributes to central systolic blood pressure is not directly proportional to blood pressure measures per se [30]. For example, studies using glyceryl trinitrate (a NO donor) have been shown to reduce measures of arterial stiffness and pulse wave reflection, independently of blood pressure changes [31]. A NO donor may reduce large vessel stiffness, even in the presence of cardiovascular risk factors including hypertension and hypercholesterolemia [31,32]. Interestingly, central measures of vascular systolic stiffness are inversely proportional to urinary measures of nitric oxide metabolism in black individuals [33]. Hence, the focus on blood pressure without an assessment of arterial stiffness parameters independently of blood pressure changes may be a missed opportunity to demonstrate the efficacy of L-arginine or L-citrulline. It is well established that central aortic stiffness is a risk factor, not only for cardiac hypertrophy, but also for coronary artery disease, dementia, and other vascular neuropathology’s [34].

Finally, we are not aware of any studies that have biochemically normalized the responses where measurements of arginase or asymmetric dimethylarginine are utilized as a primary outcome measure to identity the need to justify blood pressure responses. Firstly, arginase is the central enzyme in the urea cycle which hydrolyzes L-arginine into urea and L-ornithine [35]. Hence, the overexpression of arginase can reduce the ability of L-arginine supplementation to increase vascular nitric oxide [35]. Interestingly, translational studies have identified a tissue-specific manner in the expression of arginase with age, with a higher expression found in the lung. Thus, measuring systemic arginase as a function of age may not be useful. Secondly, normal ranges of both L-arginine and an inherent biological NOS inhibitor, asymmetric dimethylarginine (ADMA) (between 0.3–0.9 μmol/L), can saturate the eNOS enzyme, promoting NO production. Hence, under normal physiological conditions, it is proposed that additional L-arginine supplementation, irrespective of dose, would not induce eNOS activity to create more localized vascular NO [36]. In contrast, in the presence of elevated plasma concentrations of ADMA, the eNOS activity is decreased, with lower levels of vascular and NO production, meaning L-arginine may influence NO production [36].

### Limitations

In our screening of the registered clinical trials, we chose to restrict populations without specific chronic diseases, such as renal disease sub-groups. In doing so, our aims were to assess the trial designs relevant to the general population for blood pressure reduction, with or without evidence for endothelial function improvement. This, however, did not preclude those with general cardiovascular risk factors or metabolic syndrome, whereby hypertension is a common sub-component. Interestingly, when reviewing current systematic reviews on L-arginine and blood pressure, we noted that these reviews also contained studies we would have excluded or in fact did exclude, such as pregnancy. This meta-analysis we reviewed concluded that there was considerable heterogeneity between studies; once again, this is not surprising, given the lack of studies including the general population without specific diseases. Given the large volume of studies omitted based on our pragmatic exclusion criteria for blood pressure in the general population, we cannot generalize whether improvements in study design could be useful to improve heterogeneity between different disease sub-groups, sexes, or age groups. As blood pressure can be resistant to multiple therapeutic interventions, certain drug classes, and can present issues with compliance, there is a higher need for more well-designed trials including biochemical biomarkers of the regulation of L-arginine and NOS inhibition. Finally, we are cautious not to overstate our findings, given the small number of studies that were available for assessment. We suggest revisiting clinicaltrials.gov to assess future studies and their degree of variability.

We have previously suggested that studies in systematic reviews presented heterogeneity between individual RCTs; in part, this is influenced by non-standardized clinical end points. To improve heterogeneity between studies in systematic reviews and meta-analyses, protocol designs in clinicaltrials.gov need standardized inclusion and exclusion criteria for blood pressure reduction studies using the general population. We further recommend that future RCT designs ideally test interventions in community populations, and these interventions include standardized methodology for systolic and diastolic blood pressure ranges for inclusion and efficacy. Additionally, sample sizes and statistical power calculations are provided for non-pragmatic studies. Any therapeutic intervention of L-arginine should include dosage, duration, and the time of delivery. Databases such as clinical.trials.gov are intended to serve as an independent primary resource with which to assess clinical trial designs, and this is important if they are the only source of information, for example, if they have not been previously published as a full trial outcome or the protocols have not yet been published.

## 4. Materials and Methods

### 4.1. RCT Identification, Screening, and Eligibility

The U.S. Library of Medicine registry for clinical trials (https://www.clinicaltrials.gov/) was used to identify RCT for hypertension with a L-arginine and or L-Citrulline chronic intervention. A search was conducted on the 4th of November 2020 based on the search terms ‘L-Arginine or L-Citrulline and blood pressure’ or ‘L-Arginine or L-Citrulline and Hypertension’, with the inclusion parameters ‘completed trials’ and ‘Adult (18–64)’. This search strategy resulted in a pool of 44 registered RCT trials (Figure 1). These initial 44 trials were then evaluated in detail against inclusion and exclusion criteria. The inclusion criteria were based on the presence of changes in blood pressure/hypertension as a measured outcome with at least one arm of the trial. Blood pressure changes could be either a primary or secondary outcome, or could simply be listed in the trial design. The exclusion criteria included the presence of specific chronic conditions (for example, advanced renal disease) and/or acute interventions. An acute intervention is defined as not allowing sufficient time to observe the ongoing resetting of blood pressure to a lower level. Additionally, only studies that included L-arginine or L-Citrulline as a single therapeutic intervention (e.g., combined with other therapeutic agents or herbal interventions known to influence blood pressure). After applying the inclusion/exclusion criteria, five of the forty-four registered clinical trials were retained for analysis.

### 4.2. Comparison of Trial Elements

Key elements of the trial designs were assessed from the five RCTs included in the analysis. The variables for RTC design comparison are the timing of BP measures, the type of BP measure (combinations of systolic, diastolic, ambulatory, brachial, ankle, and central BP), L-Arginine/L-Citrulline dosage (concentration/volume), chronic pharmacological dosing (interval timing of dosage), inclusion and exclusion criteria based on demographic composition, and the baseline BP range. The intent of this comparison is to identify the heterogeneity of the study population and trial design.

### 4.3. Comparison of Clinicaltrials.gov Trial Elements versus Those Included in Three Recent Published Meta-Analyses

A review of PubMed with the same search terms we used in the RCT database for blood pressure reduction revealed three recent meta-analyses for L-Arginine [16] or L-Citrulline [17,18]. These meta-analyses were used to contrast clinical design elements with those published in the trials selected from clinicaltrials.gov. Additionally, we compared whether the meta-analyses had included all the published RCTs that we had identified. Furthermore, we were also interested in whether the selection of papers that were incorporated into the meta-analyses used similar inclusion/exclusion criteria as the meta-analysis itself.

### 4.4. Statistics

The extracted data were then collated, tabulated, and narratively synthesized. The data are summarized as either means, ranges, or percentages.

## 5. Conclusions

Inconsistencies in clinical trial designs in complementary medicine have been previously reported [36]. In this study, we conclude that there is a lack of consistent trial designs to understand the role of L-arginine and L-citrulline on blood pressure reduction in community adult populations. Indeed, conclusions drawn from human studies have been inconsistent between and within studies in published systematic reviews with and without meta-analysis. Published systematic reviews may have had some selection bias. We believe that inconsistent study design can be attributed to the heterogeneity of the study outcomes. Inconsistency across trial designs included differences in dosing, the duration of the L-arginine treatment, a lack of information regarding L-citrulline dosing, a lack of target ranges for diastolic and systolic blood pressure, the type of the sample population, variability in age ranges, and the role of sex in trial designs. Inconsistencies in study design for RCTs for blood pressure interventions pose a challenge for systematic reviews and meta-analysis studies in accurately assessing the effect size and impact of L-arginine on blood pressure in community settings. Until further RCT evidence is available to support L-arginine and blood pressure reduction, the use of translational animal models may continue to largely guide the evidence base.

## Figures and Tables

**Table 1:** Summary of clinical trial characteristics.

NCT Number	n (Study Population Size)	Inclusion Criteria				Exclusion Criteria	L-Arginine/L-Citrulline		BP Measure		
		Gender	Age Range (Years)	Systolic BP (mmHg)	Diastolic BP (mmHg)	Population	L-Arginine/ L-Citrulline Dosage (Per Dose)	Interval	Outcome Measures	Type of BP Measures	Timing/Interval of Measures
NCT03433963	118	Female/ Male	50–75	120–160	65–100	Cardiovascular Diseases/Coagulopathy Neurological Diseases/Pulmonary Diseases Gastrointestinal Diseases/Liver or Kidney Diseases/Cancer	L-Arg supplement 3 g	3 times/day for 2 weeks	Primary: Systolic and diastolic BP/ambulatory BP. Secondary: Endothelial nitric oxide synthase/Plasma L-Arg or L-citrulline and ornithine in L-arg metabolic pathway	Systolic and diastolic BP/Ambulatory BP	Baseline twice on the 14th day, and end point on 15th day
NCT02214290	16	Male	18–40	<160	<99	Cardiovascular Diseases	L-citrulline capsule 0.75 g	8 times/day for 1 week	Primary: Brachial, ankle, and central BP. Secondary: Aeriel Stiffness (PWV) and wave reflection	Brachial, ankle, and central BP	Baseline and end point at 1 week
NCT00137124	120	Female/ Male	18–65	Not Specified	Not Specified	Cardiovascular Diseases/Neurological Diseases/Liver or Kidney Diseases	Not Specified	Not Specified	Primary: L-arginine transport and metabolism on endothelial function	Not Specified	4 week timeframe, interval not specified
NCT02392767	25	Female/ Male	40–65	130–149	Not Specified	Cardiovascular Diseases/Underweight/Obese	L-arginine 2.4 g	2 times/day for 4 weeks	Primary: Endothelial Function. Secondary: Systolic and diastolic BP/Asymmetric dimethyl arginine (ADMA) Level. Other: Prothrombin Time	Systolic and diastolic BP	Daily during the 4th (final) week
NCT02143817	60	Female	50–70	Not Specified	Not Specified	Diabetes	L-citrulline supplementation 3 g	2 times/day for 8 weeks	Primary: Brachial and aortic BP. Secondary: Arterial Stiffness and pressure wave reflection/Endothelial and autonomic functions. Other: Body Composition/Muscle Strength	Brachial and aortic BP	8 week timeframe, interval not specified

## Data Availability

Data is contained within the article.
